# Novel real-time tumor-contouring method using deep learning to prevent mistracking in X-ray fluoroscopy

**DOI:** 10.1007/s12194-017-0435-0

**Published:** 2017-12-28

**Authors:** Toshiyuki Terunuma, Aoi Tokui, Takeji Sakae

**Affiliations:** 1 Faculty of Medicine, University of Tsukuba, Ten-nohdai 1-1-1, Tsukuba, 305-8575 Japan; 2 Proton Medical Research Center (PMRC), University of Tsukuba Hospital, Amakubo 2-1-1, Tsukuba, 305-8576 Japan; 3 Graduate School of Comprehensive Human Science, University of Tsukuba, Ten-nohdai 1-1-1, Tsukuba, 305-8575 Japan

**Keywords:** Tumor contouring, Markerless tumor tracking, Supervised deep learning, Image recognition, Data augmentation, X-ray fluoroscopy

## Abstract

Robustness to obstacles is the most important factor necessary to achieve accurate tumor tracking without fiducial markers. Some high-density structures, such as bone, are enhanced on X-ray fluoroscopic images, which cause tumor mistracking. Tumor tracking should be performed by controlling “importance recognition”: the understanding that soft-tissue is an important tracking feature and bone structure is unimportant. We propose a new real-time tumor-contouring method that uses deep learning with importance recognition control. The novelty of the proposed method is the combination of the devised random overlay method and supervised deep learning to induce the recognition of structures in tumor contouring as important or unimportant. This method can be used for tumor contouring because it uses deep learning to perform image segmentation. Our results from a simulated fluoroscopy model showed accurate tracking of a low-visibility tumor with an error of approximately 1 mm, even if enhanced bone structure acted as an obstacle. A high similarity of approximately 0.95 on the Jaccard index was observed between the segmented and ground truth tumor regions. A short processing time of 25 ms was achieved. The results of this simulated fluoroscopy model support the feasibility of robust real-time tumor contouring with fluoroscopy. Further studies using clinical fluoroscopy are highly anticipated.

## Introduction

Several motion-management techniques have been developed to irradiate targets that move due to respiratory motion, such as lung and liver tumors. The first breakthrough in this field was the respiratory-gating irradiation method, which used a patient’s external respiratory signal [1]. The second breakthrough was the development of a real-time tumor-tracking method that uses X-ray fluoroscopy [2, 3]. This method can deliver accurate irradiation because it directly detects fiducial markers implanted near a tumor. However, this method has some problems: marker implantation is invasive [4], markers produce metal artifacts on computed tomography (CT) images that result in treatment-planning errors [5], and markers locally disturb the dose profile by interacting with the treatment beam [6, 7].

Many studies of the tumor-tracking method without fiducial markers have been reported [8–24]. However, to our knowledge, these methods are not often used in clinical practice. This is because these methods are more prone to mistracking compared to methods that use fiducial markers. In many cases, the mistracking is caused by the projected bone structures; bones are enhanced as obstacles in fluoroscopic images due to a high Z-dependence on the photoelectronic effect, the primary interaction with kilovoltage (kV) X-rays. Tracking methods that use bone-suppressed fluoroscopic images have been reported recently [15–17]. These bone-suppressed images were generated using a dual-energy fluoroscopy system [15, 16] or by a special software using an artificial neural network (ANN) [17]. Since this method suppressed the obstacle features in images, improved tracking accuracy could be expected. However, we believe that the bone-suppression method is unnecessary for tumor tracking if other methods can directly recognize important and unimportant features for tracking, as that occurs in human image recognition. In other words, we can achieve robust tumor tracking by controlling computer object recognition of the tumor as important and bone as unimportant. We define this as “importance recognition control”.

Recently, deep learning has been advanced as a high-performance technique for image recognition and image segmentation [25–28]. This technique may enable markerless tumor tracking with minimal mistracking due to obstacles. Additionally, since the reported image segmentation method can detect the object’s shape in images using pixel-level classification [26, 28], it may achieve real-time markerless tumor “contouring”, unlike conventional simple tracking.

Deep learning is categorized as a data-driven optimization method. Hence, an adequate training dataset should be used to apply the method to real-time markerless tumor contouring. In radiotherapy, three-dimensional (3D) or four-dimensional (4D) CT imaging is conducted in advance for treatment planning. The deep-learning training should use these patient-specific CT data, because the data contain the individual features of the tumor, surrounding tissues, and bone structures. There is no reason to use other patients’ data for training, unlike general medical applications such as computer-aided diagnosis (CAD). However, as deep learning requires at least several hundred training data points [25–28], preparing a training dataset from one specific patient has been difficult until now. For example, only 10 digitally reconstructed radiographs (DRRs) can be obtained from standard respiratory-phase 4D CT data. These training datasets are too small to meet the requirements of effective deep learning. This difficulty is the “data augmentation” problem in the research field of deep learning.

Accordingly, to achieve markerless tumor contouring using deep learning, we must determine how to control importance recognition and how to increase the available training data. Here, we briefly explain our new strategy to solve those problems. Although a detailed understanding of deep learning is difficult, the essence of deep learning in image recognition can be regarded as the detection of common features from a large number of images using co-occurrence probability. For example, supervised deep learning, the method used to train a dataset using both training data and ground-truth data, detects some co-occurring features between the two datasets. Here, it can be hypothesized that different co-occurrence probabilities induce different importance recognition. For example, if a target feature in training images is located at the same position of ground-truth features in supervised images, this strong positional relationship may induce the recognition that this feature is “important.” In contrast, if an obstacle is located randomly at an incorrect position in a large number of training images, this positional decorrelation between training images and supervised images may induce the recognition that this feature is “unimportant”. In this paper, we call this devised method the “random overlay method.” This method can be applied for the markerless tumor tracking because the feature of tumor as target and that of bone structures as obstacles can be separated using patient-specific 3D CT data obtained before treatment planning. In addition, as treatment planning has already been completed, the projected image of a gross tumor volume (GTV) or a clinical tumor volume (CTV) may be used as the supervised image for deep-learning training. The random overlay method will also solve the data augmentation problem because the method easily enlarges the training images.

In this study, we propose a real-time tumor-contouring method that uses deep learning to prevent mistracking caused by obstacles. This method is based on the hypothesis that a new random overlay method of data augmentation induces the opposite importance recognition by deep learning. The purpose of this study was to prove this hypothesis and verify the accuracy of tumor contouring with minimal mistracking caused by bone structures using simulated X-ray fluoroscopic images.

## Methods

### Overview of workflow

**Fig. 1:**
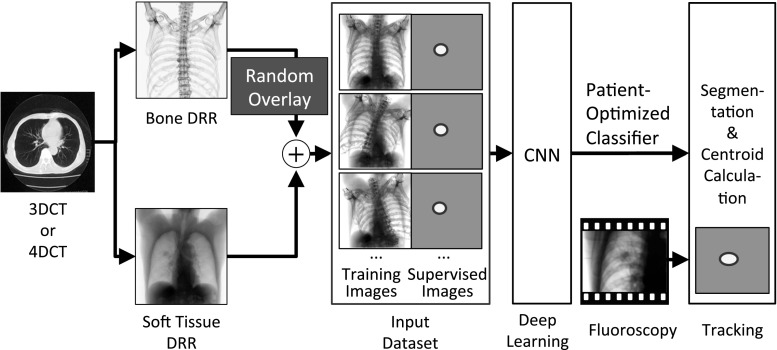
Overall workflow of the proposed tumor-contouring method. *3DCT* three-dimensional computed tomography, *4DCT* four-dimensional CT, *DRR* digitally reconstructed radiograph, *CNN* convolutional neural network

This section focuses on the overall workflow and aims of the method. The overall workflow of the proposed real-time tumor-contouring method using deep learning with importance recognition control is shown in Fig. 1.

First, we assume that a target and some surrounding important objects can be separated from an obstacle. When tracking a tumor, a soft-tissue DRR and a bone-structure DRR were separately created by projecting patient-specific 3D CT data after selecting an appropriate threshold for the CT value; details of the DRR and threshold will be provided in Sect. 2.3.2. Second, we generated a large number of random overlaid images as training images for deep learning from the soft-tissue and randomly arranged bone-structure DRRs. This method can create the large number of training images required for effective training of deep learning from the 3D CT data of a single patient. Simultaneously, the projected tumor region was segmented as a region of interest (ROI) in supervised images. For example, the projected GTV or CTV was appropriate in a segmented region in clinical use. The mathematical expressions of a pair of images are as follows:

1 $$I_{\text{training}} \left( {x , y} \right)\; = \;I_{\text{soft}} \left( {x , y} \right) \;+\;I_{\text{bone}} \left( {x + \delta_{x} ,\;y +\delta_{y} } \right)$$

2 $$I_{\text{supervised}} \left( {x , y} \right)\; =\;\left\{ {\begin{array}{*{20}c} { 1 \;({\text{target}}) } \\ { 0\; ({\text{others}})} \\ \end{array} } \right.,$$

where *I*_soft_, *I*_bone_, *I*_training_, and *I*_supervised_ are the soft-tissue DRR, bone-structure DRR, training image, and supervised image, respectively, *δ*_*x*_ and *δ*_*y*_ are random integers that independently shift the bone-structure DRR in the *x* and *y* directions, respectively.

Next, more than 1000 pairs of training and supervised images were processed using a convolutional neural network (CNN). The CNN could generate an individually optimized classifier after deep-learning training. Finally, from this classifier and a fluoroscopic image, we were able to obtain an output image in which all pixels were classified as “tumor” or “not tumor,” as shown in Fig. 1. This pixel classification was identical to the tumor contouring. The tumor position was calculated as the centroid in this output image.

As mentioned in the introduction, the purpose of the random overlay method is to control importance recognition using different co-occurrence probability of features between training images and supervised images. As the tumor region in the training images was the same as the ROI in the supervised images, a strong positional correlation existed between them. However, no positional correlation existed between the bone structures in the training images and the ROIs in the supervised images of the random overlay method. Thus, as deep-learning training proceeded, we could expect that this strong correlation, or the absence of a correlation, automatically created different levels of importance recognition for tracking.

### Details of deep learning and image segmentation

**Fig. 2:**
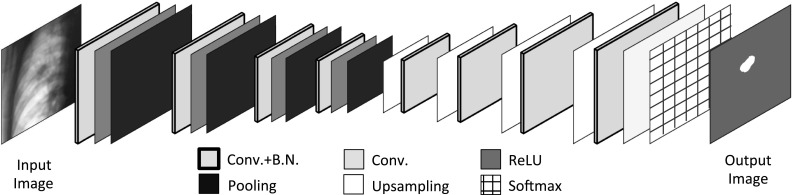
CNN architecture of supervised deep learning. The CNN is composed of four encode and four decode processes. The total number of layers is 30. *CNN* convolutional neural network, *Conv* convolution layer, *BN* batch normalization layer, *ReLU* activation function layer

The CNN calculations in this study were performed using a computer (Linux OS: Ubuntu 16.04; CPU: Xeon E5649, Intel Corp., CA, USA; memory: 48 GB) with a dual graphics processing unit (GPU; GeForce GTX 1080, NVIDIA Corp., CA, USA) and the deep-learning frameworks Caffe [25] and SegNet [26]. Although the main applications of SegNet are object recognition and image segmentation for self-driving cars, some medical applications of both SegNet and Caffe have been reported [27, 28]. The actual layer architecture of the CNN used in this study is shown in Fig. 2.

The CNN was composed of four encode processes, four decode processes, and a softmax layer. The total number of layers was 30. The kernel size of the convolution layers was 7 × 7. The pooling and upsampling amplitudes were 1/2 × 1/2 and 2 × 2, respectively. These parameters were essentially the same as those described by Kendall [26]. The encode process executed extraction and abstraction of the object features by reducing the image size, and the decode process restored the image size. The final softmax layer classified all pixels as “tumor” or “not tumor.” The tumor region was identified in the output image as the segmented region, as shown in Fig. 2.

### Models

We evaluated the robustness and accuracy of our proposed tumor-contouring method using the following geometric and simulated fluoroscopy models.

#### Geometric model

A preliminary validation test was performed to confirm that this method could segment a target region accurately even if the target was partially hidden by an obstacle. The workflow of this validation was the same as the workflow in Fig. 1; however, the training images, supervised images, and test images were different.

**Fig. 3:**
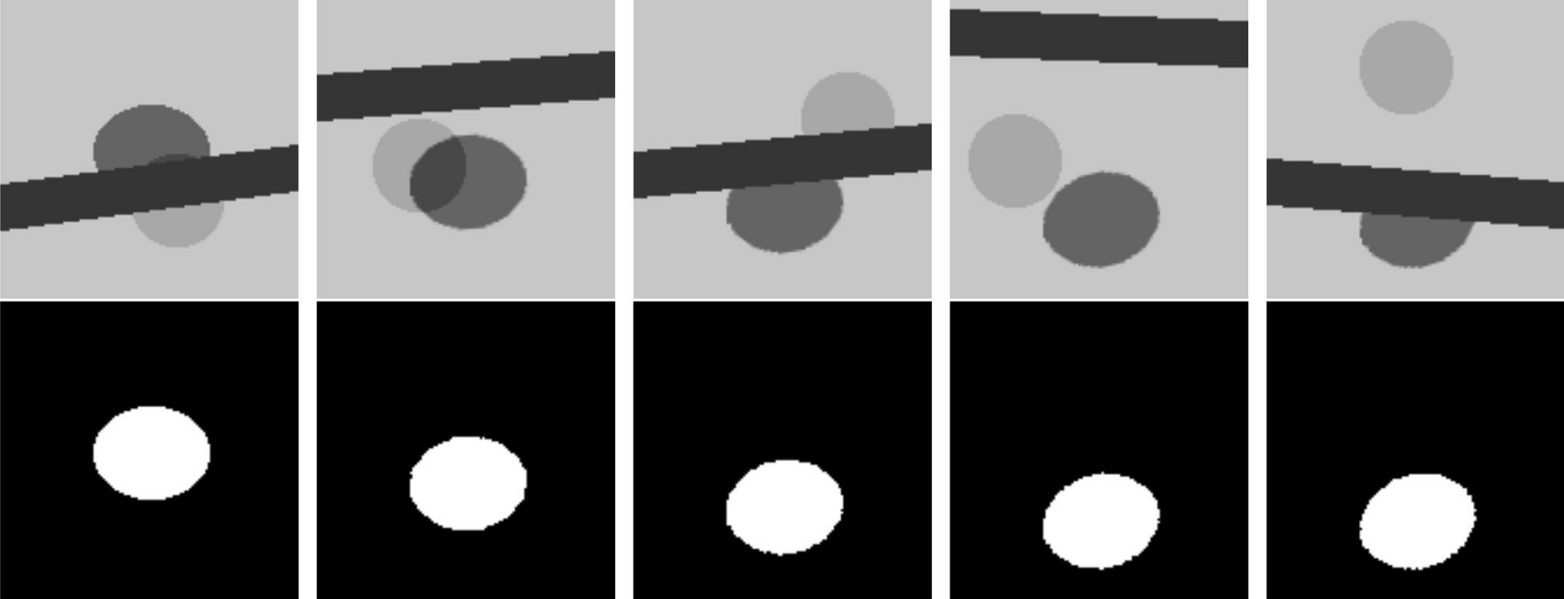
Five examples of training data. The training dataset consists of pairs of a training image (upper) and a supervised image (lower). The training images were created from a target (ellipse) and randomly overlapped obstacles (bold line and circle). The supervised images indicate the target position and shape

Examples of the arrangement of objects are shown in Fig. 3. In this geometric model, the image size and pixel depth were 128 × 128 pixels and 8-bit, respectively. The ellipses with a pixel value of 128 were substitutes for the target tumor, and the bold lines with a pixel value of 0 were substitutes for bone structure. The pixel value of other areas was 255. The ellipse was shifted and rotated using a sine-wave-like trajectory in the vertical (*y* axis) direction as a simple simulation of tumor motion and deformation due to respiration. The amplitude of the sine-wave-like motion was 30 pixels, peak-to-peak. The obstacle bold line was overlapped randomly on the ellipses and partially hid their shape. The range of the random arrangement of the bold line in both sides of the right and left frames was ± 50 pixels. Additionally, a circle with a pixel value of 64 was placed randomly with a range of ± 50 pixels to create a severe condition that induced mistracking. At the same time, the supervised images were generated as binary images, which indicated the region of the ellipse. In this manner, the total combination of object arrangements in the image was greater than 2 billion. We randomly generated 2300 pairs of model and supervised images. Next, these pairs were separated into two groups to execute cross-validation. The group consisting of the first 2000 pairs was used for training; a second group consisting of the final 300 pairs was used for testing. As the total image variation was in excess of 2 billion, there was very little overlap between the training and test images.

To train this model using deep learning, the training dataset consisting of 2000 pairs of images was processed by the CNN. The CNN created an optimized classifier. To examine the performance of this classifier, the 300 model images of the test dataset were individually entered into the optimized classifier. Finally, the 300 output images that indicated the target region were generated. These 300 output images were compared to the corresponding 300 supervised images that indicated the target’s actual location and shape.

#### Simulated fluoroscopy model

The simulated fluoroscopy model was tested to validate the proposed tumor-contouring method. The test was not performed directly using clinical fluoroscopy because it is currently very difficult to verify tumor contours in all clinical fluoroscopy frames; thus, it is difficult to evaluate the accuracy of the tumor contouring.

The workflow of this validation was the same as the workflow illustrated in Fig. 1. We randomly selected the 3D CT data of four patients with lung cancer in different sites (upper right, middle right, lower right, and lower left). These individual 3D CT data were obtained using exhalation gating by CT (Optima CT580W, General Electric Company, Connecticut, USA) and a respiratory-gating system (AZ-733V, Anzai Medical Co., Tokyo, Japan). The original resolution and size of the CT images were 1.07 × 1.07 × 2.5 mm and 512 × 512 × (patient-specific slice number) in the left–right (LR), anterior–posterior (AP), and superior–inferior (SI) directions, respectively. To define bone structures as obstacles, we classified all pixels of CT images into two groups according to a threshold of 200 Hounsfield units (HU). This threshold was selected because in all patients, the CT values of ribs, which have the lowest bone density, were greater than approximately 250 HU and the CT values of soft tissue including the tumor were less than approximately 60 HU. The soft-tissue and bone-structure DRRs were obtained individually by separately accumulating these two groups of CT data in the AP direction with bicubic interpolation. Next, a partial image of the DRR was extracted to fit the imaging field of the actual X-ray fluoroscope, which is approximately 300 × 300 mm. The final resolution and size of the DRRs were 1.5 × 1.5 mm and 256 × 256 pixels, respectively.

**Fig. 4:**
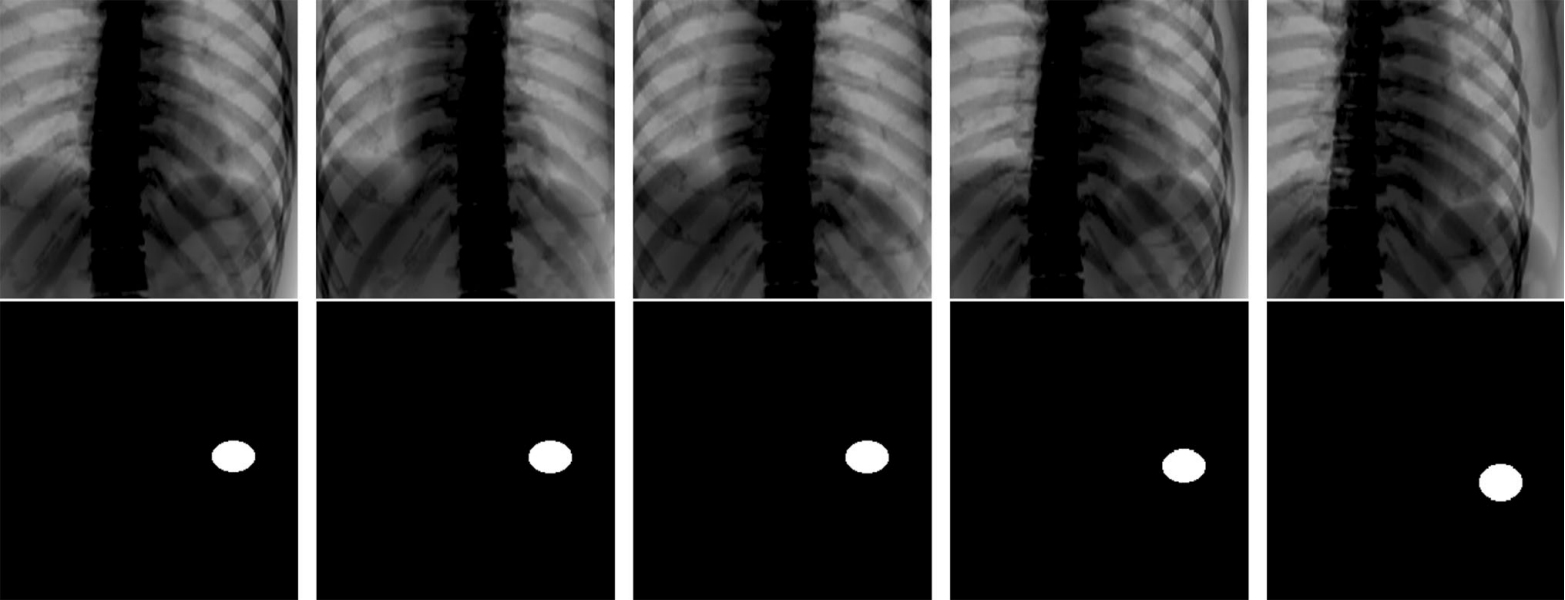
Five examples of training data for patient 4. The training dataset consists of pairs of a training image (upper) and a supervised image (lower). The training images were created from soft-tissue DRR and randomly overlapped bone DRR. The supervised images indicate the tumor position and shape

We then prepared 2000 pairs of training and supervised images of each patient. The training images were generated by overlapping the randomly arranged bone-structure DRR on the soft-tissue DRR. Examples of a pair of training and supervised images are shown in Fig. 4. In this model, tumor movement and deformation due to respiration were simulated by expanding the soft-tissue DRR in the SI direction to the fourth power of the sine of amplitude* α*_*i*_. The mathematical expressions of the training image are as follows:

3 $$I_{\text{training}} \left( {x , y} \right) \;= \;I_{\text{soft}} \left( {x , \alpha_{i} y} \right) \;+\; 2\;I_{\text{bone}} \left( {x + \delta_{x} ,\; y + \delta_{y} } \right)$$

4 $$\alpha_{i} \;{ = 1 \;/\; }\{ {1.1\;+\; 0.1 \;{ \sin }^{4} (i/45)} \}$$

5 $$- 1 0 \;{ < }\;\delta_{x} < 1 0 ,\; - 1 0\; { < }\;\delta_{y} < 1 0 ,$$

where *i* is the frame index. The pixel value of the bone-structure DRR was doubled to increase the tracking difficulty. The range of the random arrangement was ± 10 pixels, which corresponded to ± 15 mm in both image directions. The final training images were normalized as 8-bit images. The supervised images were generated by segmenting the tumor region as an ellipse from the respiratory-expanded soft-tissue DRRs. Finally, we obtained the individually and automatically optimized classifier after deep learning training using the individual input dataset.

In the examination, we prepared 300 pairs of test and supervised images for each patient. The test images were the simulated fluoroscopic images generated using the expanded soft-tissue and fixed bone-structure DRRs without using the random overlay method. The mathematical expression of test image *I*_test_ is as follows:

6 $$I_{\text{test}} \left( {x , y} \right) \;= \;I_{\text{soft}} \left( {x , \alpha_{i} y} \right) \;+\; 2\;I_{\text{bone}} \left( {x , y} \right)$$

Here, the probability of a test image coinciding with a training image was 1/400, because the range of random bone arrangements in the training image was ± 10 pixels. Thus, 99.7% of the test images and training images were different. The supervised images were generated to learn the true segmentations using the same method as the training process. These 300 test images were processed by the individually optimized classifier and output images were generated.

### Evaluation methods

The similarity between the segmented tumor regions in the output images using the proposed method and the true regions in the supervised images was calculated using the Jaccard index *J*:

6 $$J = \frac{S \cap T}{S \cup T} ,$$

where *S* is the segmented tumor region and *T* is the “true” region. The tumor positions identified by the proposed method were calculated as the centroid of the segmented tumor region in the output images. The tracking error was calculated by comparing it with the “true” tumor position. The correlation coefficient *R* between this tumor trajectory and the “true” trajectory was also calculated.

The tracking error and correlation coefficient of the trajectories were also calculated using the results of the conventional template-matching method; the normalized cross-correlation (NCC) algorithm was used to compare its accuracy with our proposed method. The template images were manually selected as rectangular regions that included the targets in the exhalation-phase images.

## Results

### Geometric model

**Fig. 5:**
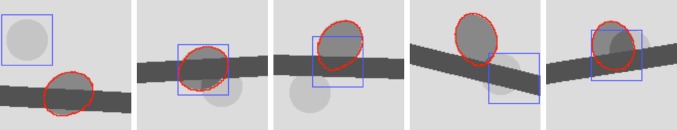
Example of the tracking results for the geometric model. The red circles show the result of segmentation using the proposed method; the blue squares show the target position tracked by the template-matching method

**Fig. 6:**
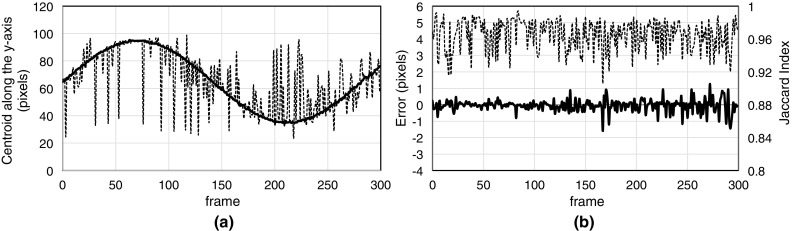
**a** Centroid trajectory of the segmented region according to the proposed method (bold line), and the trajectory of the center position obtained using the template-matching method (dashed line). **b** The tracking error (bold line) and Jaccard index (dashed line) according to the proposed method

The processing times of our method were 10 min for training and 7 ms/frame for contouring and tracking. Five sample images among 300 frames are shown in Fig. 5. The contour lines of the target segmented using our method are drawn in red, and the tracked positions of the template-matching method are drawn as blue squares. The results of the tracking trajectories of each method are shown in Fig. 6a, and the tracking error and accuracy of segmentation using our method are shown in Fig. 6b. A statistical summary of the tracking error, the correlation between the tracked trajectory and the ground truth, and the similarity between the segmented result and true value are listed in Table 1.

**Table 1:** Summary of the results of the geometric model

	Proposed method	Template matching
Error (pixel)	− 0.01 ± 0.43 (SD)	0.13 ± 15.4 (SD)
Correlation	0.999	0.727
Jaccard Index	0.966 (0.906–0.994)	–

The results of segmented images in Fig. 5 show that our method could detect a nearly perfect shape of the ellipse targets even if the targets were partially hidden by obstacles. As summarized in Table 1, accurate segmentation with a similarity of approximately 0.96 according to the Jaccard index and accurate tracking within an error of ± 0.5 pixels were achieved. Thus, these results clearly showed that the proposed target-contouring method prevented mistracking caused by obstacles. The hypothesis that the random overlay method controls importance recognition was confirmed by these results.

### Simulated fluoroscopy model

**Fig. 7:**
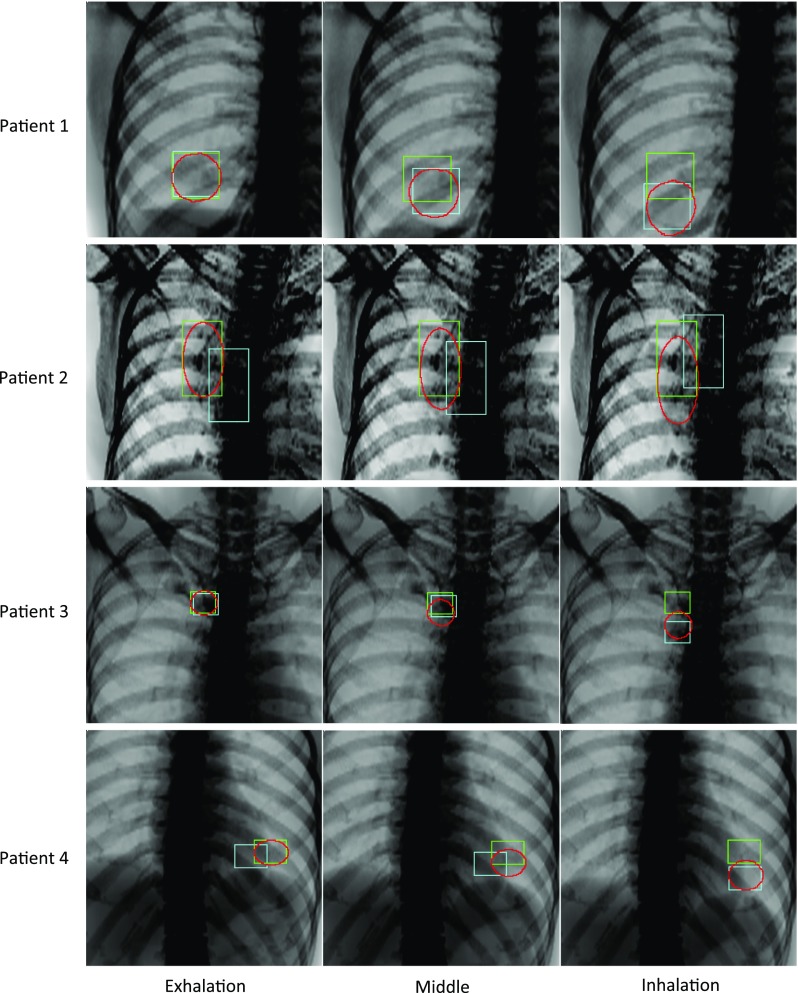
Tracking results for simulated fluoroscopy. The red contours show the segmented tumor region according to the proposed method. The green and blue squares show the results of the template-matching method using the regular DRR (soft-tissue + bone) and the soft-tissue DRR templates, respectively

**Fig. 8:**
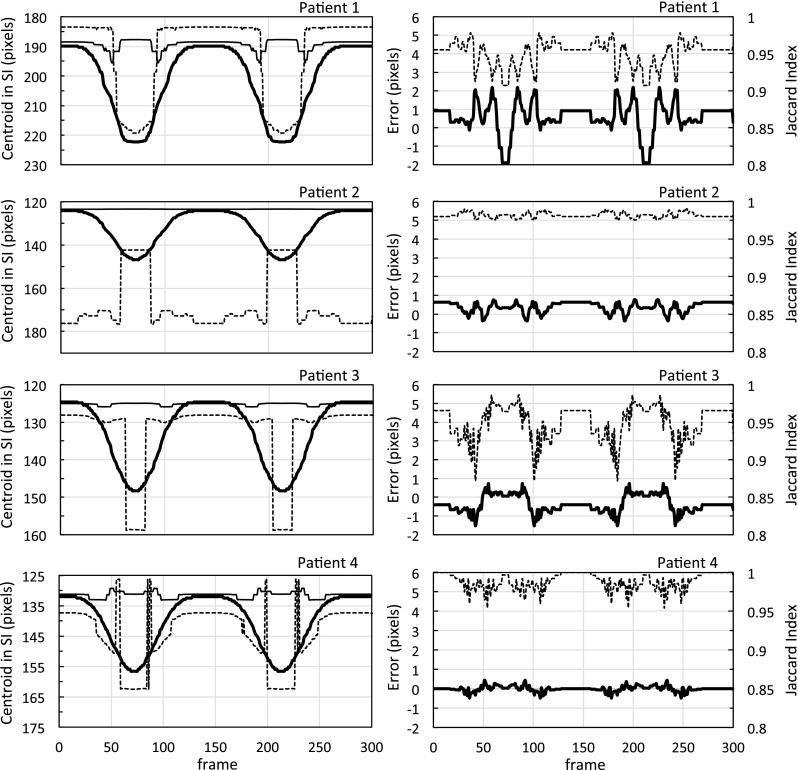
The images on the left show the trajectories of the centroid of the segmented region according to the proposed method (bold line), and the trajectories of the center position according to the template-matching method using the regular DRR template (normal line) and the soft-tissue DRR template (dashed line). The images on the right show the tracking error (bold line) and the Jaccard index (dashed line) according to the proposed method

The processing times of our method were 90 min for training and 25 ms/frame for contouring and tracking. Sample images of the result at three respiration phases (exhalation, inhalation, and middle) among 300 result images for each patient are shown in Fig. 7. The red contours show the results of the segmented tumor region using our method. The green and blue squares show the results of the template-matching method using the regular DRR template (soft-tissue and bone) and the soft-tissue DRR template, respectively. The tumor trajectories calculated by each method are shown in the left side of Fig. 8, and the tracking error and accuracy of segmentation according to the Jaccard index are shown on the right. A statistical summary of the tracking error, the correlation between the calculated tumor trajectory by our method and the ground truth, and the similarity between the segmented result and the ground truth are listed in Table 2.

**Table 2:** Summary of the results of the virtual fluoroscopy model

Method		Patient 1	Patient 2	Patient 3	Patient 4
Proposed method	Error (mm)	0.87 ± 1.2	0.56 ± 0.42	− 0.35 ± 0.70	− 0.01 ± 0.22
	Correlation	0.998	0.999	0.999	0.999
	Jaccard Index	0.950 (0.907–0.978)	0.982 (0.975–0.990)	0.949 (0.872–0.986)	0.988 (0.954–1.000)
Template matching (soft)	Error (mm)	− 13 ± 9.0	55 ± 30	− 12 ± 11	6.0 ± 9.1
	Correlation	0.910	− 0.800	0.703	0.790
Template matching (soft + bone)	Error (mm)	− 18 ± 19	− 6.1 ± 12	− 0.69 ± 13	− 13 ± 14
	Correlation	0.090	− 0.969	− 0.078	− 0.339

Error (mm) was converted according to the relationship 1.5 mm = 1 pixel

Figure 8 shows that, of the detected tumor trajectories, conventional template-matching using the NCC algorithm failed to detect the correct positions of the tumor with simulated respiratory motion. In contrast, the proposed method detected the tumor at approximately the same position as the ground truth. From the statistical results in Table 2, the tracking error of the proposed method was approximately within 1 mm. The correlation coefficient between the tracked and ground truth centroids was greater than 0.998 in all cases. In addition, a high similarity of approximately 0.95 according to the Jaccard index was demonstrated between the segmented tumor region and the ground truth. These results confirmed that the proposed method was accurate and prevented mistracking caused by bone structure.

## Discussion

This report presents a real-time tumor-contouring method that used deep learning to prevent mistracking. The novelty is the data augmentation method; a random overlay method was used to control differences in importance recognition. This method is based on the hypothesis that different positional correlations of features between training and supervised images induce different importance recognition in image recognition by deep learning. Using this method, we can prepare a large number of training images and conduct effective deep-learning training. Since training images are completely associated with the patient, the generated classifier by deep learning is also optimized for that patient. Although a detailed understanding of how this deep-learning method computes image segmentation is difficult, the results prove four advantages of this method.

The first and most important advantage of this method is the prevention mistracking caused by obstacles. Despite it was difficult to track the tumor in the test image because the enhanced bone structures were overlapped as obstacles, our method achieved accurate tumor contouring of over 0.95 according to the Jaccard index, and accurate tumor tracking with an error of approximately 1 mm. These results prove clearly that our method prevents mistracking caused by bone structures. This robustness can be achieved by recognizing that bone features are unimportant for tracking. Consequently, our hypothesis, that the different positional correlation of features induces different importance recognition in deep learning, is justified. Compared with other studies, the tracking accuracy within an error of approximately 1 mm using our method is approximately the same as or superior to bone-suppression methods [15–17]. It is also comparable to other results of multi-phase template-matching methods that subtract respiratory-phase images to improve tumor-motion enhancement and bone-feature suppression [10–12]. Additionally, our method will effectively minimize mistracking caused by other obstacles, such as a treatment couch frame projected onto fluoroscopic images at a non-zero projection angle [21, 22].

The second advantage of this method is the prevention mistracking due to low-visibility tumors. In previous tumor tracking methods using multi-region template-matching [13, 14], the tracked positions of a few manually selected, clearer features compensated for the tracking uncertainty. In contrast, our method does not require manual selection of some features near the tumor. As all soft-tissue features in training images have a strong positional correlation with the ground truth in supervised images, following this hypothesis, it is reasonable to consider that all soft-tissue features are recognized as important. We can presume that all soft-tissue features in the fluoroscopic image assist with image segmentation. Indeed, in spite of the low-visibility tumors in Patients 2 and 3, our results demonstrated accurate contouring. In Patient 1, the worst tracking result was observed during inhalation. It is believed that the diaphragm extended outside the image and disappeared during inhalation. This was caused by inadequate simulations that placed the tumor and diaphragm near the edge of the virtual fluoroscopy image. However, this unfavorable result was evidence that our proposed method identified the tumor region using not only the tumor features, but also features of the surrounding structures. These facts strengthen the evidence in support of our hypothesis.

The third advantage of the proposed method is that it provides tumor contouring. Although many studies have examined tumor tracking and not tumor contouring [10–22], our method can provide tumor contouring because the CNN performs pixel-level classification. Our method has a strong advantage because tumor contouring will lead to real-time adaptive radiotherapy. Currently, we can compare our results only with those of Zhang [23], who tracked tumor boundaries. The similarity of our results, which were approximately 0.95 according to the Jaccard index, are comparable to those of Zhang’s method [23]. However, a detailed comparison is difficult because the test models are different.

The fourth advantage of the proposed method is real-time processing. The short processing time necessary for tumor contouring, approximately 25 ms/frame, is superior to the 500 ms/frame reported by Zhang [23]. Here, we define “real time” as the achievement of a short processing time of 33 or 66 ms, corresponding to a specification of 30 or 15 frames/s in general X-ray fluoroscopy. The duration of a typical pulsed X-ray irradiation in a fluoroscopy system is less than 4 ms as reported by Shirato [2]. Thus, it seems reasonable that our method, with a 25-ms processing time, will achieve real-time processing of 30 frames/s although additional processes, such as data transfer between the fluoroscopy system and the computer, require less than 4 ms.

We understand that our results were obtained from preliminary simulated fluoroscopic images, and we must validate this method using real clinical fluoroscopy. The anticipated primary difficulty is the different image qualities between the DRRs and the clinical fluoroscopy images. However, we expect that this problem can be solved by improving the DRR quality to be similar to the quality of clinical fluoroscopy images, or by creating a wide contrast variation in the training images for the input dataset of deep learning. We consider that this proposed method is valuable in principle and is a breakthrough in markerless tumor tracking.

## Conclusions

We have proposed a real-time, markerless, tumor-contouring method using deep learning to prevent mistracking caused by bone structures on X-ray fluoroscopy. The novelty of our method is the combination of the devised random overlay method and supervised deep learning. The expected effect of the method was to induce importance recognition for tracking: the understanding that soft-tissue features are important and bone is unimportant. From the results of the simulated fluoroscopy model, high-speed and accurate tumor contouring can be achieved even if a low-visibility tumor and a strong bone structure are visible on fluoroscopy. Therefore, the successful effects of this method of real-time tumor contouring have been proven. Further studies to validate the effectiveness of this proposed method in clinical fluoroscopy are essential.
